# Salivary Immunoglobulin a Alterations in Health and Disease: A Bibliometric Analysis of Diagnostic Trends from 2009 to 2024

**DOI:** 10.3390/antib13040098

**Published:** 2024-11-29

**Authors:** Jakub Jankowski, Kacper Nijakowski

**Affiliations:** Department of Conservative Dentistry and Endodontics, Poznan University of Medical Sciences, 60-812 Poznan, Poland; jjankowski41@wp.pl

**Keywords:** saliva, IgA, diagnostics, marker, health, disease, stress, exercise

## Abstract

Background/Objectives: Salivary immunoglobulin A (IgA) is a mediator of local immunity and host defence. Altered IgA levels may predispose to bacterial invasion of the mucosa in the gastrointestinal tract, including the oral cavity. Our study aimed to present the diagnostic trends related to salivary IgA in health and disease based on a bibliometric analysis of published papers between 2009 and 2024. Methods: By 14 September 2024, 1247 English original articles were found in the database Web of Science. We selected 838 records considering the diagnostic usefulness of IgA in human subjects. Based on bibliographic data, we created citation and keyword co-occurrence maps using VOSviewer 1.6.20. Results: Most articles belonged to the “Sport Sciences” category (*n* = 169), followed by the “Immunology” category (*n* = 93). The Brazilian researcher Alexandre Moreira from the University of Sao Paulo had the most published and most frequently cited papers. Most of the included articles came from the USA (*n* = 158), England (*n* = 105), Brazil (*n* = 95), and Japan (*n* = 95). The most cited article described research on IgA in response to SARS-CoV-2 infection (*n* = 690), but the subsequent two papers considered the role of salivary IgA in the dysbiosis of the intestinal microbiota in inflammatory bowel diseases (*n* = 272) and the formation of systemic immune responses from the gastrointestinal tract (*n* = 245). Conclusions: Salivary IgA is a widely evaluated diagnostic marker in both patients and healthy individuals. Numerous reports have identified its changes as a result of physical exertion in various groups of athletes, during infections (including SARS-CoV-2) and in the course of local diseases (e.g., periodontal disease) or systemic diseases (e.g., inflammatory bowel disease).

## 1. Introduction

Salivary immunoglobulin A plays a pivotal role in mucosal immunity, acting as the predominant immunoglobulin within mucus secretions, including saliva, where it serves as a key biomarker of immune function [1,2]. Salivary IgA is produced by B-lymphocytes adjacent to mucosal cells and transported across epithelial cells via the polymeric immunoglobulin receptor (pIgR) before being released into the oral environment [3,4]. This process is crucial for maintaining mucosal immunity in various body regions, including the respiratory and gastrointestinal tracts.

The production and secretion of salivary IgA are influenced by neuroendocrine factors, particularly during periods of acute stress, when sympathetic nervous system activation can enhance both antibody release and the translocation of IgA across epithelial barriers [5,6]. This dynamic regulation highlights the complex interplay between psychological stressors and immune function, making salivary IgA a promising biomarker for stress-related changes in immune responses.

Furthermore, salivary IgA plays a vital role in protecting against pathogen invasion at mucosal surfaces. Its ability to neutralise toxins, inhibit colonisation by microorganisms, and prevent the entry of pathogens into systemic circulation underscores its importance in both oral health and systemic immunity [6,7]. Notably, salivary IgA is under circadian control, with levels peaking in the morning and gradually declining throughout the day. This suggests potential synchrony with cortisol levels and highlights the need for precise timing in salivary IgA measurement [8,9].

Salivary IgA is also implicated in age-related immune changes. Levels of IgA in saliva are lower in infants, gradually increasing throughout childhood and adolescence, reaching adult levels by around 20 years of age [10,11]. However, salivary IgA production decreases with age, contributing to an increased risk of infection in the elderly population [12]. This decline in salivary IgA production, coupled with age-related changes in the gut microbiome, suggests that mucosal immunity is particularly vulnerable in older adults, necessitating further research into strategies to bolster salivary IgA levels in this demographic [13,14,15].

Given the ease of saliva collection and the role of IgA in mucosal immunity, salivary IgA has emerged as a valuable biomarker for various health conditions, including respiratory infections and stress-related immune suppression [2]. Its potential as a diagnostic tool, particularly in field settings such as military environments or during intensive training, has been well-documented [16,17,18]. Moreover, selective IgA deficiency or reduced salivary flow has been linked to a higher incidence of infections, further emphasising the diagnostic significance of salivary IgA levels [19,20].

The present study aims to explore diagnostic trends related to salivary IgA in health and disease through a bibliometric analysis of published research from 2009 to 2024. The bibliometric analysis allows to identify the most impactful publications, presenting the current state of a study field and the most trending research topics. By examining patterns in the literature, this analysis will provide insights into the evolving understanding of salivary IgA as a biomarker and its applications across various medical fields.

## 2. Materials and Methods

The search was carried out using the Web of Science database belonging to Clarivate. The following search formula was used: TS = ((saliva* AND (“immunoglobulin A” OR “IgA”)) AND (diagnostic OR marker OR health OR disease OR stress OR exercise)). We applied the filters of the years of publication from 2009 to 2024, the type of publication as an original article, and the English language.

By 14 September 2024, 1247 records were found in the database. We selected 838 papers that met the additional inclusion criterion, which was diagnostic usefulness in human subjects, excluding in vitro and animal studies. Two researchers made the selection based on titles, abstracts, and sometimes full texts.

Data on selected records were taken directly from the Web of Science database and imported for analysis in VOSviewer 1.6.20 (a bibliometric software program from the Centre for Science and Technology Studies of Leiden University). Based on bibliographic data, we created citation and keyword co-occurrence maps. In the map, the bubble size demonstrated the number of publications, while the distance between them demonstrated their relatedness. In the network visualisation, the same-coloured bubbles formed clusters, indicating close collaboration. Detailed information is available in the figure legends.

## 3. Results

**Figure 1:**
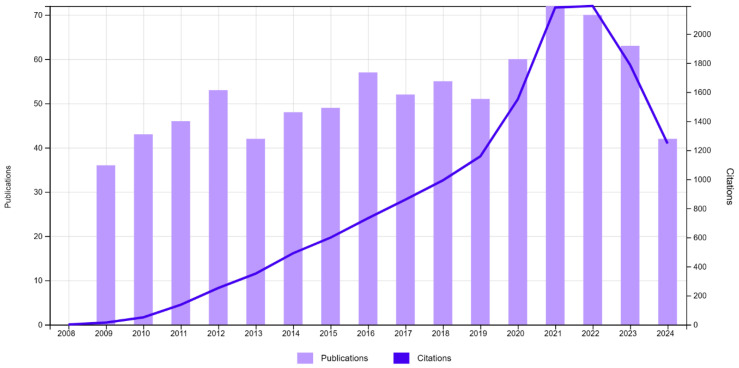
Number of publications and their citations between 2009 and 2024 (Web of Science).

The number of included publications with their citations is presented in Figure 1.

**Figure 2:**
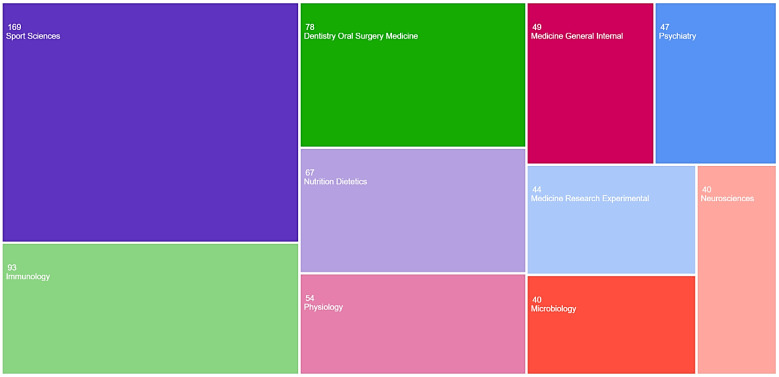
TreeMap chart visualisation for the top 10 Web of Science categories.

Most articles belonged to the “Sport Sciences” category (*n* = 169, 20.1%), followed by the “Immunology” category (*n* = 93, 11.1%). The first ten most common categories according to WoS are presented in Figure 2.

**Figure 3:**
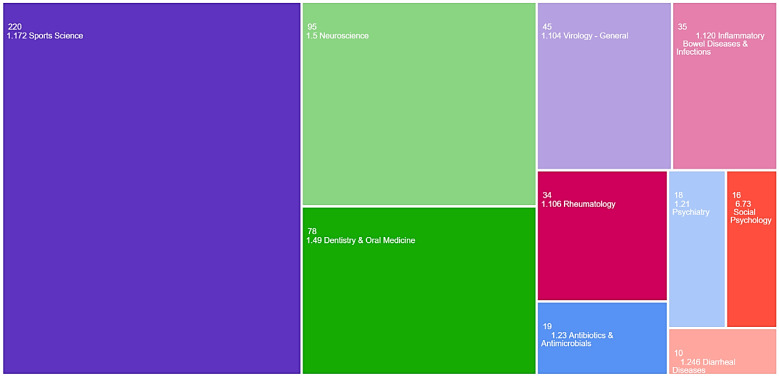
TreeMap chart visualisation for the top 10 meso citation topics (Web of Science).

In the meso citation topics analysis, most records were classified as “1.172 Sports Science” (*n* = 220, 26.2%), “1.5 Neuroscience” (*n* = 95, 11.3%), and “1.49 Dentistry and Oral Medicine” (*n* = 78, 9.3%)—Figure 3.

**Figure 4:**
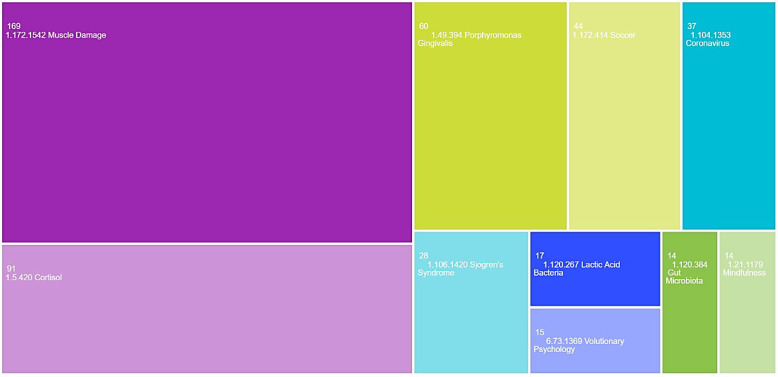
TreeMap chart visualisation for the top 10 micro citation topics (Web of Science).

However, in the micro citation topics analysis, the term “1.172.1542 Muscle Damage” dominated (*n* = 169, 20.1%), as shown in Figure 4.

Table 1 shows publishers who have published at least 25 papers. Elsevier was the leader with the number of 137 articles, which constituted 16.3% of all the papers included in the analysis.

Table 2 lists the journals that most frequently published papers on salivary IgA and whose papers had the most total citations. In both rankings, “*Journal of Strength and Conditioning Research*” (Lippincott Williams & Wilkins, Impact Factor 2.5) was in first place.

**Figure 5:**
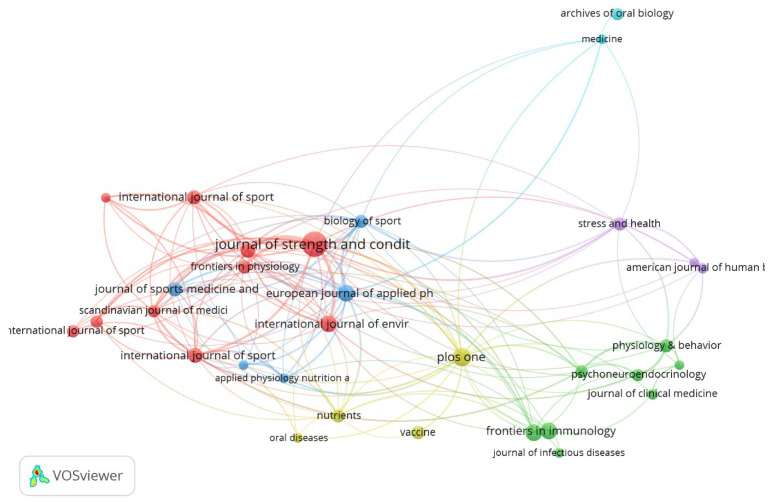
The network visualisation for journals with minimum 5 publications based on citation analysis.

Figure 5 illustrates the network of citation connections between journals. “*Journal of Strength and Conditioning Research*” had the noticeably highest total link strength (106).

Considering the authors’ contributions, Alexandre Moreira (University of Sao Paulo, H-index 33, 121 publications, 2853 citations) had the most published and most frequently cited papers—Table 3.

**Figure 6:**
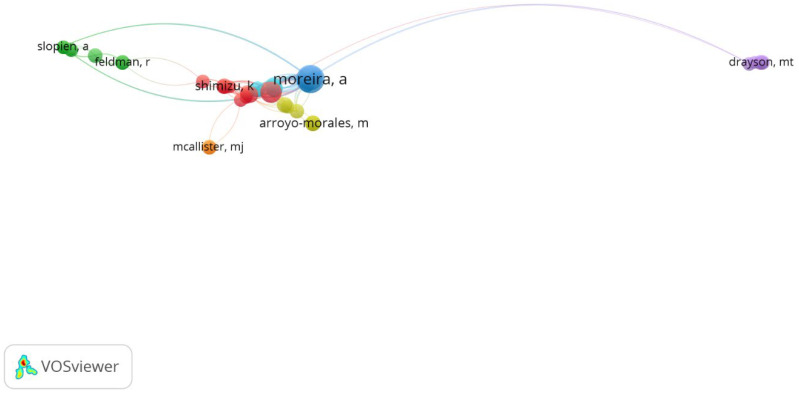
The network visualisation for authors with minimum 5 publications based on citation analysis.

The network of connections between the authors is shown in Figure 6, where most of the clusters are connected to A. Moreira (total link strength 259), as expected.

Eleven centres had at least ten publications. The leader in the ranking was the University of Sao Paulo (Brazil). Similarly, the centres with the most total citations are presented. Linkoping University (Sweden) is worthy of attention, as it has as many as 393 citations with only six published works. Detailed data are shown in Table 4.

**Figure 7:**
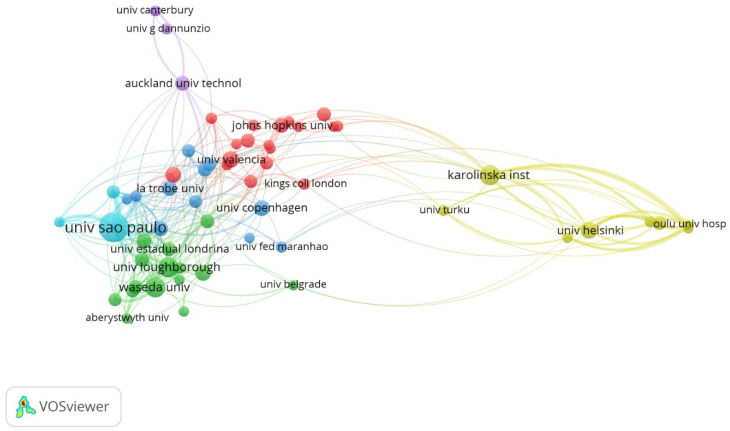
The network visualisation for organisations with minimum 5 publications based on citation analysis.

Most centres were associated with the University of Sao Paulo (Brazil; total link strength 174) and then Loughborough University (England; total link strength 84). The secondary yellow cluster was concentrated around two centres: the University of Helsinki (Finland; total link strength 85) and Karolinska Institute (Sweden; total link strength 81), as reported in Figure 7.

Most included articles came from the USA (*n* = 158, 18.8%) and England (*n* = 105, 12.5%). Table 5 shows countries that published at least 25 articles on salivary IgA.

**Figure 8:**
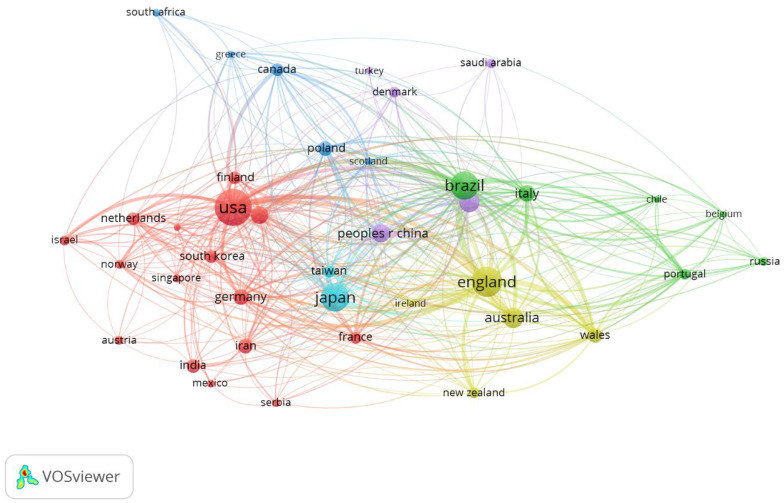
The network visualisation for countries with minimum 5 publications based on citation analysis.

The three most frequently cited countries included the USA (3406 citations, total link strength 542), England (2415 citations, total link strength 717), and Brazil (1697 citations, total link strength 557)—Figure 8.

Table 6 presents the 20 most common keywords in the included records—the top rankings were “saliva”, “IgA”, and “cortisol”.

**Figure 9:**
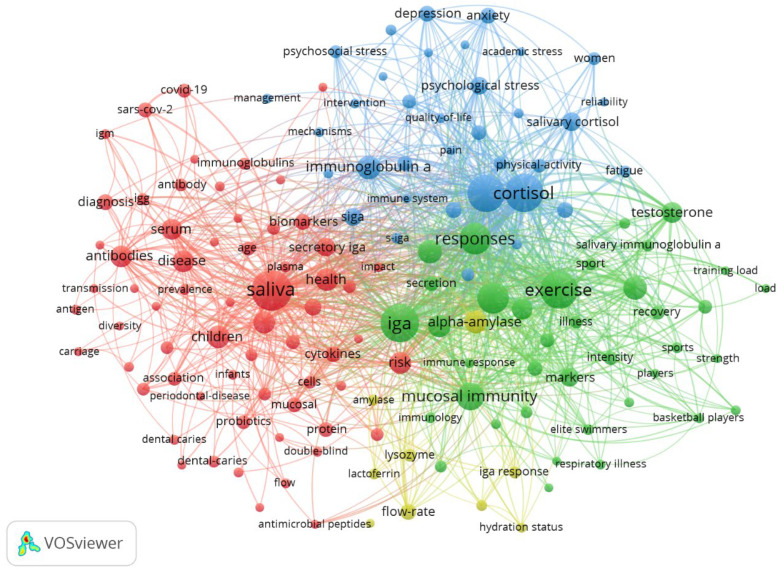
The network visualisation for keywords with minimum 10 occurrences based on co-occurrence analysis.

**Figure 10:**
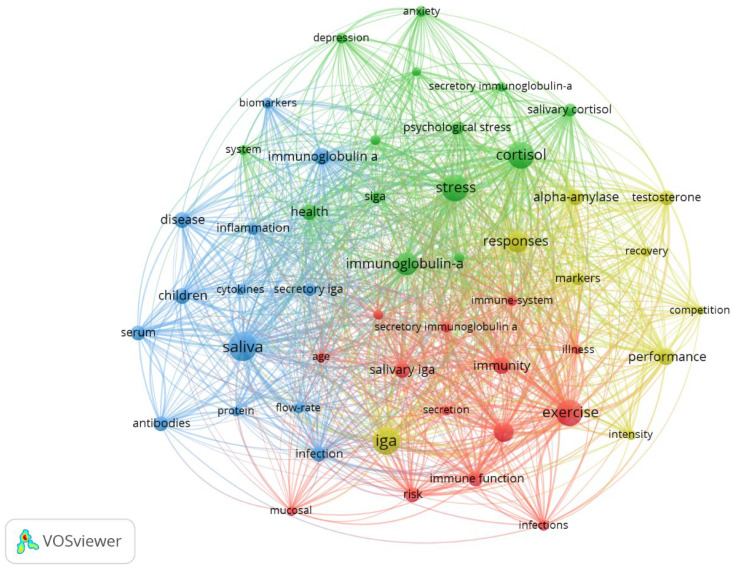
The network visualisation for the top 50 keywords based on co-occurrence analysis.

In the context of keyword co-occurrence, two analyses of network relationships were considered: for all keywords occurring at least ten times (Figure 9) and for the top 50 keywords (Figure 10). Based on the first analysis, clear clusters can be observed regarding physical activity and sports (green), stress and mental health (blue), and immunological and inflammatory processes in the pathogenesis of local and systemic diseases (red). In general, the five keywords with the strongest links coincide with the five most common ones: cortisol (total link strength 791), stress (total link strength 776), exercise (total link strength 765), IgA (total link strength 694), and saliva (total link strength 639)—values are given for the second analysis.

Table 7 shows the most frequently cited articles assessing salivary IgA levels—records with at least 75 citations were included. The most cited article concerned IgA in response to SARS-CoV-2 infection (Sterlin et al., 2021 [21]; *n* = 690). Interestingly, the next two papers on the podium considered the role of salivary IgA in the dysbiosis of the intestinal microbiota in inflammatory bowel diseases (Said et al., 2014 [22]; *n* = 272) and in the formation of systemic immune responses from the gastrointestinal tract (Sjögren et al., 2009 [23]; *n* = 245).

## 4. Discussion

The bibliometric analysis enables us to identify potential gaps and observed trends in the state of the art of the research field. Eight hundred and thirty-eight papers that described the diagnostic utility of IgA in humans were selected from the records indexed in the Web of Science during the analysed period. Most of the papers on this topic were published between 2021 and 2022. Thus, the highest numbers of citations were also observed in these two years. The three most productive publishers were Elsevier, Springer Nature, and Wiley. On the other hand, the most productive and most cited journal was “*Journal of Strength and Conditioning Research*” from the publisher Lippincott Williams & Wilkins.

The most published papers and the most citations comprised the achievements of the Brazilian researcher Alexandre Moreira from the University of Sao Paulo, which was also the centre with the most publications and citations. Brazil ranked third in the most productive countries, behind the United States and England. Among the most cited papers, unsurprisingly, was the article on IgA in SARS-CoV-2 infection, but the top three also included two papers in the field of gastroenterology.

The bibliometric analysis can initiate impactful future research addressing essential societal issues. On the basis of the above results, we decided to discuss the diagnostic trends regarding salivary IgA in the fields of medicine most considered in the last 15 years. Sport sciences, immunology, oral medicine, gastroenterology, and psychiatry were among the most frequently discussed research areas, and these were also reflected in the cluster analysis of the most common keywords.

### 4.1. IgA in Sport Sciences

Salivary immunoglobulin A is a key mediator of mucosal immunity, playing a critical role in protecting athletes from upper respiratory tract infections (URTIs), particularly during periods of intensified physical training. A substantial body of research has demonstrated a strong association between reductions in salivary IgA levels and an increased risk of URTIs, underscoring the importance of salivary IgA in inhibiting viral replication and bacterial adhesion at mucosal surfaces [36]. This relationship has been consistently observed across multiple sports disciplines, including endurance activities, high-intensity intermittent exercise, and resistance training, where diminished salivary IgA levels during or following intensive exercise have been linked to a heightened susceptibility to infection [20,37,38,39].

Prolonged or intense exercise has been shown to induce transient reductions in salivary IgA secretion, aligning with the “open window theory,” which postulates a period of immune suppression post-exercise that renders athletes more vulnerable to infections [40]. This phenomenon is particularly evident in endurance athletes, where lower pre-exercise baseline salivary IgA levels correlate with an increased incidence of upper respiratory symptoms (URSs). For instance, recreational endurance runners reporting URSs during a structured training intervention exhibited significantly lower salivary IgA concentrations both before and after the training period, highlighting salivary IgA as a potential biomarker for assessing URTI risk [41].

Despite the well-documented association between salivary IgA and URTI susceptibility, the predictive utility of IgA levels in saliva appears to vary across athletic populations. Studies such as those by Cunniffe et al. failed to identify a significant correlation between individual salivary IgA concentrations and URTI occurrence in rugby players, suggesting that while salivary IgA serves as a valuable marker of immune function, its diagnostic value may be context-dependent [42]. Nonetheless, the majority of evidence supports a clear relationship between reduced salivary IgA secretion rates and increased infection risk, particularly during periods of acute physical or psychological stress, such as competition days, where elevated cortisol levels have been implicated in the suppression of salivary IgA production [42,43].

The interplay between salivary IgA and cytokine regulation further elucidates the complexity of mucosal immunity in athletes. Studies have shown that athletes with higher susceptibility to URTIs often exhibit reduced salivary IgA secretion alongside elevated levels of interleukin-10 (IL-10), an immunosuppressive cytokine known to inhibit salivary IgA production [29]. This suggests that the regulation of IgA in saliva may be influenced by broader immunological processes, emphasising the need for interventions to maintain salivary IgA levels during periods of intense training. Strategies such as nutritional supplementation and stress management techniques may offer potential avenues for bolstering mucosal immunity and mitigating the risk of infection in athletes during peak training periods.

### 4.2. IgA in Immunology

Salivary IgA plays a crucial role in mucosal immunity, and recent studies have highlighted its significance in various immunological contexts, particularly concerning SARS-CoV-2 infections, vaccination responses, and microbiome interactions.

A substantial body of research has focused on salivary IgA in relation to SARS-CoV-2 infection. It has been shown that IgA is the dominant early antibody response in saliva, where mucosal-associated lymphocytes locally produce it. Importantly, salivary IgA specific to SARS-CoV-2 remains detectable longer after symptom onset (49 to 73 days post-symptoms) than blood IgA, which is detectable for about a month [21]. These findings are of significant importance in light of growing evidence regarding the types of antibodies linked to effective protection against reinfection and the potential need for vaccine strategies to focus on inducing a robust, though potentially transient, IgA response [21]. Moreover, elevated IgA levels in saliva correlate with the severity of COVID-19, underscoring its role in immune responses during infection [44].

Detecting anti-SARS-CoV-2 IgA in saliva offers a significant advantage for diagnostic purposes, particularly in point-of-care testing [45]. Saliva collection is non-invasive and easier to perform, making it an ideal method for testing in both low-resource settings and vulnerable populations, such as the elderly and infants. Studies have further confirmed that salivary IgA levels correlate well with serum IgA, reinforcing its utility as a diagnostic marker [26]. This has led to widespread interest in using salivary IgA as part of diagnostic and screening strategies for COVID-19 [28].

Seth et al. demonstrated that sublingual vaccination against Group A Streptococcus (GAS), using virus-like particles, elicited high levels of IgA in saliva [46]. In the context of vaccination, particularly against SARS-CoV-2, studies have demonstrated that prior exposure to the virus significantly enhances the salivary IgA response post-vaccination. Individuals with a history of COVID-19 infection exhibited higher salivary IgA titres following mRNA vaccination compared to seronegative individuals [34]. This suggests a potential synergistic effect of pre-existing immunity in amplifying mucosal responses after vaccination, which may be a crucial factor to consider in developing future vaccine formulations targeting SARS-CoV-2 and other respiratory pathogens.

Moreover, the early microbiome has been shown to influence IgA levels. Early-life colonisation by specific gut microbiota, such as Bifidobacterium species, has been associated with higher salivary IgA levels, suggesting that the microbiome may play a critical role in shaping mucosal immunity during infancy. This finding opens new avenues for researching how gut health and microbiota composition could be modulated to enhance IgA-mediated protection against infections and allergies [23].

In conclusion, the collective body of research demonstrates the significant role of salivary IgA in immune defence, particularly at mucosal surfaces. Its involvement in responses to pathogens like SARS-CoV-2, its potential for use in diagnostics, and its response to vaccination highlight its critical function in mucosal immunity. Further research is necessary to fully understand the mechanisms driving IgA responses, particularly in individuals without prior mucosal immunity.

### 4.3. IgA in Oral Medicine

Numerous studies have addressed the role of salivary IgA in antimicrobial defence, highlighting its ability to inhibit microbial adhesion to both mucosal and dental surfaces, as well as its capacity to facilitate the removal of microorganisms such as *Streptococcus mutans*, a key pathogen in dental caries, through agglutination. Consequently, salivary immunoglobulins work synergistically with innate immune mechanisms, and the production of salivary antibodies, particularly against pathogens like streptococci, plays a crucial role in the early colonisation of oral surfaces [3,47,48,49,50,51].

Additionally, several studies have reported a lower incidence of dental caries in individuals with higher concentrations of salivary IgA. Based on these findings, the researchers concluded that secretory IgA in saliva plays a protective role in preventing the development of dental caries [49,52,53]. Also, a significant positive correlation was observed between salivary IgA levels and the DMFT index in patients with uncontrolled type 2 diabetes [54].

Moreover, IgA levels indicate broader immune competence in various oral conditions, particularly in immunocompromised individuals. Mandal et al. highlight that HIV-infected individuals, who often exhibit lower salivary IgA levels due to immune suppression, are more prone to dental caries. The study underscores that reduced IgA production, a consequence of diminished CD4+ T-helper cell function, directly correlates with increased caries prevalence in these patients [49]. These findings suggest that IgA concentration in saliva may be a valuable biomarker for oral diseases like dental caries and for assessing immune function in systemic conditions such as HIV.

Higher levels of salivary IgA were observed in oral lichen planus [55]. In the context of burning mouth syndrome (BMS), studies have shown conflicting results regarding salivary IgA concentrations. For example, one study reported a significant decrease in salivary IgA secretion rates among BMS patients, alongside reduced salivary flow, suggesting a compromised salivary defence mechanism [56]. In contrast, Lopez-Jornet et al. found elevated salivary IgA levels in BMS patients, possibly due to the smaller volume of saliva rather than increased production [57]. Similar conclusions to those of Lopez-Jornet et al.’s studies have been reported previously [58,59,60]. These opposing findings highlight the complexity of salivary IgA dynamics in BMS and suggest that alterations in IgA levels in saliva may be influenced by changes in salivary flow rather than immune response alone. Further research is required to elucidate the mechanisms underlying these discrepancies.

Other reports indicate that salivary IgA’s role extends to fungal infections, particularly candidiasis. Salivary IgA can coat the cell walls of *Candida albicans*, inhibiting its adhesion to mucosal surfaces [50]. Specific salivary IgAs have been identified to react with antigens, such as heat shock mannoproteins, which are crucial for controlling *Candida* growth. This protective mechanism is further supported by findings from Hibino et al., who reported that saliva from Candida-free individuals significantly inhibited the growth of blastoconidia, the infectious form of *C. albicans* [61]. This points to a potential role for salivary IgA in maintaining oral fungal homeostasis and preventing candidal overgrowth, particularly in immunocompromised or otherwise susceptible individuals.

Collectively, these studies emphasise the diagnostic potential of salivary IgA in oral medicine, particularly in identifying susceptibility to oral diseases such as dental caries, periodontitis, candidiasis, and conditions associated with altered immune status like HIV. The variability in salivary IgA levels across different pathologies also highlights its potential as a non-invasive biomarker for both local and systemic health conditions.

### 4.4. IgA in Gastroenterology

Studies in gastroenterology concerning salivary IgA have predominantly focused on its role in inflammatory bowel disease (IBD). Notably, the two primary forms of IBD—Crohn’s disease (CD) and ulcerative colitis (UC)—have demonstrated distinct immunological profiles, particularly in response to biologic therapies [62]. According to Nijakowski et al., UC patients who responded positively to biologic treatment exhibited a significant eight-fold increase in salivary IgA, while no such increase was observed in CD patients. Furthermore, this IgA elevation was correlated with a reduction in UC severity, as measured by the Mayo scale, indicating an improvement in oral mucosal immunity alongside therapeutic success in UC patients [62]. However, it remains unclear why this effect is not replicated in CD patients.

Contrasting findings from several other studies suggest that increased salivary IgA levels have been observed in active IBD cases, including both UC and CD [22,63,64]. This discrepancy may be attributed to the presence of abnormalities in the oral mucosa in these studies, which were not present in the patient cohort examined by Nijakowski et al. [22,63,64]. Moreover, Said et al. propose that elevated IgA levels in IBD may be a response to dysbiosis of the oral microbiota, reflecting systemic immune activation linked to concurrent gut microbiota alterations [22]. This highlights a potential connection between oral and intestinal immune responses in the pathogenesis of IBD.

Moreover, Janšáková et al. indicates the potential role of salivary IgA as a biomarker in gastroenterology, particularly for the early diagnosis and monitoring of CD and orofacial granulomatosis (OFG) [64]. Elevated levels of salivary IgA in patients with CD and OFG, along with increased concentrations of other immune markers such as lactoferrin (LF), indicate an enhanced mucosal immune response. Notably, this occurs despite the absence of a significant rise in myeloperoxidase (MPO), a traditional marker of inflammation. These findings suggest that salivary IgA could be employed as a non-invasive biomarker to assess mucosal immune function and disease progression in patients with CD and OFG.

Additionally, Saluja et al. demonstrated that elevated levels of the IgA1 and IgA2 subclasses were observed in patients during both flare-up and remission phases of recurrent aphthous ulceration, a condition often associated with CD [65,66,67]. This further underscores the relevance of salivary IgA as a key marker of mucosal immunity in gastrointestinal disorders. Similarly, Aase et al. emphasise that salivary IgA levels are linked to the activation of intestinal immune responses, reinforcing its potential utility in the diagnosis and monitoring of IBD [68].

### 4.5. IgA in Psychiatry

Physiological stress systems and the immune system are functionally linked [69]. Cortisol, a steroid hormone secreted in response to stress, is commonly used to assess the activity of the hypothalamic–pituitary–adrenal (HPA) axis and has been extensively studied in the context of mental stress physiology [70,71]. Elevated cortisol levels may decrease IgA levels, thereby weakening immune defences; however, this relationship and the underlying psychological processes remain unclear and require further investigation [69,72].

The authors have focused on various stress factors, such as ship-borne journeys and Antarctic environments as stress situations, loneliness, depression, and academic stress (defined as prolonged examination periods), and their impact on salivary IgA levels. Stressful situations, anxiety attacks, depression, and other psychological conditions can elevate cortisol levels in the body. Engeland et al. found that higher perceived stress is associated with lower levels of salivary IgA1, but not IgA2. Additionally, their findings suggest that stress directly influences secretory immunity by modulating IgA secretion by B cells. Engeland et al. further pointed out that loneliness and depression may indirectly affect secretory immunity by altering the salivary IgA transport mechanism [5]. Moreover, numerous authors have previously demonstrated that secretory IgA release is under strong neuroendocrine control, and acute stress studies have consistently shown robust effects on salivary IgA, typically resulting in increased concentrations [6,73,74].

Another study concluded that stressful conditions such as ship-borne journeys and the Antarctic environment may induce changes in salivary IgA levels [75]. In contrast, Murphy et al. found that academic stress, defined as an extended examination period, did not alter salivary IgA levels in undergraduate students. The study reported increased salivary cortisol concentrations and heightened perceived stress during the examination period compared to non-examination periods, but no significant change in salivary IgA levels was observed between the two periods [72].

Watamura et al. examined the relationship between salivary cortisol concentration and antibody secretion in children across the day, both at home and in childcare, and their association with parent-reported illnesses [69]. The results indicated that elevated cortisol profiles during childcare, particularly in the afternoon, predicted lower antibody levels on the following weekend. Additionally, higher cortisol levels during the weekend were associated with greater parent-reported illness, and a declining daily pattern of salivary IgA was observed in both childcare and weekend days for older preschoolers but only on weekend days for younger preschoolers. These findings suggest that elevated cortisol levels in children during childcare may be linked to reduced antibody levels and increased illness frequency.

Researchers have also attempted to investigate the impact of other diseases, such as Autism Spectrum Disorder (ASD) and anorexia nervosa (AN), on IgA levels. Gong et al. found that salivary IgA content was significantly reduced in children with ASD and demonstrated diagnostic value for ASD [76]. This study suggests new possibilities for ASD diagnosis and the prevention of oral diseases in ASD populations, as well as provides evidence of an ASD-specific mucosal immunophenotype in the oral cavity. However, Paszyńska et al. investigated a possible correlation between opiorphin, stress/immune biomarkers such as cortisol, salivary alpha-amylase (sAA), and IgA in patients with restrictive-type anorexia nervosa (AN) [77]. AN patients showed significantly increased levels of cortisol and salivary IgA. These findings suggest that stress responses can be effectively assessed in saliva samples from AN patients, and both cortisol and IgA may be valuable markers for mental stress evaluation in saliva [42].

### 4.6. IgA in Other Disciplines

The diagnostic properties of salivary IgA have also been recognised in other medical disciplines, such as geriatrics and aerospace medicine. As immunity tends to decline with age, efforts to enhance immune function in the elderly are particularly relevant. In a study by Lefevre et al., supplementation with probiotics like *Bacillus subtilis* increased salivary IgA levels in elderly participants, suggesting that dietary interventions may strengthen mucosal immunity in this vulnerable population [33]. This finding supports the idea that maintaining or enhancing IgA responses through nutritional strategies could have therapeutic implications for improving immune defences, especially in older adults.

Moreover, saliva testing is especially beneficial in the elderly due to the sample collection’s ease and non-invasive nature. Salivary IgA has also found applications as a biomarker in the field of aviation. Wilder-Smith et al. investigated the impact of hypobaric hypoxic conditions during long-haul flights, hypothesising that such conditions might impair immune function and increase susceptibility to respiratory infections [78]. However, their findings showed that a 10 h overnight simulated flight under hypobaric hypoxic conditions did not result in significant immune impairment or abnormal levels of IgA and cortisol.

Space travel substantially challenges normal immune system function, potentially endangering crew safety and mission success in future deep-space explorations [79,80,81,82,83]. Collecting biological samples in the unique conditions of the International Space Station (ISS) can be complex and hard, making the use of saliva an attractive alternative. In a study by Agha et al., saliva samples from ISS crew members were analysed at various time points—long before flight, on the day of flight, upon return to Earth, and while in space aboard the ISS [84]. The study reported that salivary IgA, lysozyme, LL-37, and cortisol:DHEA were elevated in ISS crew members both before and during the mission compared to control subjects on Earth. Notably, many of these changes were related to flight experience, with rookie crew members displaying lower salivary IgA but higher levels of α-amylase, lysozyme, and LL-37 during and after the ISS mission compared to veteran astronauts.

### 4.7. Study Limitations

One of the main limitations of bibliometric analysis is citation bias. Papers published in high-impact journals, as well as prepared by researchers from prestigious universities, can be expected to be cited more often. The bibliometric analysis itself provides only quantitative data without a qualitative analysis of the included publications (as opposed to a systematic review). Therefore, in the case of our article, we tried additionally to qualitatively discuss the main diagnostic trends. Although a bibliometric analysis is usually limited to specific narrow disciplines, recognising the interdisciplinary nature of science, we allowed for a broader scope including the diagnostic possibilities of salivary IgA in different disciplines of medicine.

## 5. Conclusions

Based on our analysis, salivary IgA is a widely considered diagnostic biomarker in health and diseases. Numerous studies have identified its alterations as a result of physical activity in various groups of athletes, during bacterial, viral, and fungal infections (including SARS-CoV-2) and in the course of local diseases (e.g., periodontal disease) or systemic diseases (e.g., inflammatory bowel diseases). Identifying the most highly cited publications, the bibliometric analysis allowed us to highlight the currently observed trends, as well as potential gaps and possibilities for further research in this field.

## Figures and Tables

**Table 1:** Top productive publishers with minimum 25 publications.

Publishers	*n*	% of 838
Elsevier	137	16.3
Springer Nature	95	11.3
Wiley	86	10.3
MDPI	54	6.4
Lippincott Williams & Wilkins	48	5.7
Taylor & Francis	45	5.4
Frontiers Media Sa	41	4.9
Sage	26	3.1
Human Kinetics Publ Inc.	25	3.0

**Table 2:** Top 10 most productive and cited journals.

Top Productive Journals		Top Cited Journals		
Journal	Documents	Journal	Documents	Citations
*Journal of Strength and Conditioning Research*	30	*Journal of Strength and Conditioning Research*	30	893
*Plos One*	17	*International Journal of Sport Nutrition and Exercise Metabolism*	11	476
*European Journal of Applied Physiology*	14	*European Journal of Applied Physiology*	14	375
*International Journal of Environmental Research and Public Health*	14	*Plos One*	17	302
*Frontiers in Immunology*	13	*Journal of Sports Sciences*	11	264
*Scientific Reports*	13	*Scandinavian Journal of Medicine & Science In Sports*	7	213
*International Journal of Sport Nutrition and Exercise Metabolism*	11	*Brain Behavior and Immunity*	7	198
*Journal of Sports Sciences*	11	*Physiology & Behavior*	9	183
*International Journal of Sports Physiology and Performance*	10	*Stress and Health*	9	158
*Journal of Sports Medicine and Physical Fitness*	10	*Frontiers in Physiology*	9	145

**Table 3:** Top 10 productive and cited authors.

Top 10 Productive Authors		Top 10 Cited Authors		
Author	Documents	Author	Documents	Citations
Moreira, A	24	Moreira, A	24	712
Aoki, MS	18	Gleeson, M	14	668
Gleeson, M	14	Aoki, MS	18	596
Fang, SH	10	Bishop, NC	9	349
Bishop, NC	9	Granger, DA	7	266
Arroyo-Morales, M	8	Walsh, NP	8	226
Diaz-Rodriguez, L	8	Freitas, CG	6	207
Shimizu, K	8	Fang, SH	10	191
Suzuki, K	8	Davison, G	7	182
Walsh, NP	8	Chang, CK	7	178

**Table 4:** Top productive and cited organisations.

Top Productive Organisations		Top Cited Organisations		
Organisation	Documents	Organisation	Documents	Citations
Univ Sao Paulo	38	Univ Sao Paulo	38	897
Karolinska Inst	17	Univ Loughborough	17	689
Univ Loughborough	17	Karolinska Inst	17	606
Waseda Univ	17	Univ Copenhagen	11	412
Univ Helsinki	12	Linkoping Univ	6	393
Univ Valencia	12	Univ Tokyo	8	348
Univ Copenhagen	11	Johns Hopkins Univ	9	271
Univ Granada	11	Univ Estadual Campinas	10	258
Univ Tsukuba	11	Univ Fed Rio Grande Do Norte	7	249
Univ Bath	10	Univ Valencia	12	227
Univ Estadual Campinas	10	Bangor Univ	8	226

**Table 5:** Top productive countries with minimum 25 publications.

Country	*n*	% of 838
USA	158	18.9
England	105	12.5
Brazil	95	11.3
Japan	95	11.3
Australia	51	6.1
Spain	50	6.0
China	39	4.7
Sweden	35	4.2
Italy	34	4.1
Germany	29	3.5
Poland	26	3.1

**Table 6:** Top 20 keywords.

Keyword	Occurrences	Total Link Strength
saliva	197	639
IgA	186	694
cortisol	181	791
exercise	176	765
stress	175	776
immunoglobulin-a	125	509
responses	122	526
mucosal immunity	93	404
performance	73	300
immunity	71	257
immunoglobulin a	71	275
health	66	231
salivary IgA	66	233
alpha-amylase	63	285
children	63	177
disease	60	162
infection	59	223
risk	59	224
antibodies	58	135
immune function	56	261

**Table 7:** List of the 15 most-cited publications with minimum 75 citations.

Authors	Title	Journal	Year	Citations
Sterlin, D; et al. [21]	IgA dominates the early neutralising antibody response to SARS-CoV-2	*Science Translational Medicine*	2021	690
Said, HS; et al. [22]	Dysbiosis of salivary microbiota in inflammatory bowel disease and its association with oral immunological biomarkers	*DNA Research*	2014	272
Sjögren, YM; et al. [23]	Influence of early gut microbiota on the maturation of childhood mucosal and systemic immune responses	*Clinical and Experimental Allergy*	2009	245
Ogawa, Y; et al. [24]	Proteomic analysis of two types of exosomes in human whole saliva	*Biological & Pharmaceutical Bulletin*	2011	186
Gleeson, M; et al. [25]	Daily probiotic (Lactobacillus casei Shirota) reduction in infection incidence in athletes	*International Journal of Sport Nutrition and Exercise Metabolism*	2011	172
Randad, PR; et al. [26]	COVID-19 serology at population scale: SARS-CoV-2-specific antibody responses in saliva	*Journal of Clinical Microbiology*	2021	163
Cox, AJ; et al. [27]	Oral administration of the probiotic Lactobacillus fermentum VRI-003 and mucosal immunity in endurance athletes	*British Journal of Sports Medicine*	2010	151
Roda, A; et al. [28]	Dual lateral flow optical/chemiluminescence immunosensors for the rapid detection of salivary and serum IgA in patients with COVID-19 disease	*Biosensors & Bioelectronics*	2021	135
Gleeson, M; et al. [29]	Respiratory infection risk in athletes: association with antigen-stimulated IL-10 production and salivary IgA secretion	*Scandinavian Journal of Medicine & Science in Sports*	2012	124
Thorpe, R; Sunderland, C [30]	Muscle damage, endocrine, and immune marker response to a soccer match	*Journal of Strength and Conditioning Research*	2012	117
Childs, CE; et al. [31]	Xylo-oligosaccharides alone or in synbiotic combination with Bifidobacterium animalis subsp lactis induce bifidogenesis and modulate markers of immune function in healthy adults: a double-blind, placebo-controlled, randomised, factorial cross-over study	*British Journal of Nutrition*	2014	110
Aanæs, K [32]	Bacterial sinusitis can be a focus for initial lung colonisation and chronic lung infection in patients with cystic fibrosis	*Journal of Cystic Fibrosis*	2013	80
Lefevre, M; et al. [33]	Probiotic strain Bacillus subtilis CU1 stimulates immune system of elderly during common infectious disease period: a randomised, double-blind placebo-controlled study	*Immunity & Ageing*	2015	79
Sano, K; et al. [34]	SARS-CoV-2 vaccination induces mucosal antibody responses in previously infected individuals	*Nature Communications*	2022	76
Prodan, A; et al. [35]	Interindividual variation, correlations, and sex-related differences in the salivary biochemistry of young healthy adults	*European Journal of Oral Sciences*	2015	75

## Data Availability

The original contributions presented in the study are included in the article, further inquiries can be directed to the corresponding authors.
